# Concrete silo collapse: emergency medical services response to a mass casualty incident

**DOI:** 10.1186/s13049-025-01376-5

**Published:** 2025-04-07

**Authors:** Peter Martin Hansen, Marius Rehn, Rasmus Peter Jakobsen, Jesper Byrdorf, Simon Hestbech Lundorff, Søren Mikkelsen

**Affiliations:** 1 Prehospital Research Unit, Region of Southern Denmark, Kildemosevej 15, Odense C, 5000 Denmark; 2 Mobile Emergency Care Unit, Department of Anesthesiology and Intensive Care Unit, Odense University Hospital, Baagøes Allé, Svendborg, 5700 Denmark; 3 Department of Regional Research, University of Southern Denmark, Campusvej 55, Odense M, 5230 Denmark; 4 Danish Air Ambulance, Brendstrupgaardsvej 82, Aarhus N, 8200 Denmark; 5 Air Ambulance Department, Division of Prehospital Services, Oslo University Hospital, Postboks 4950 Nydalen, Oslo, 0424 Norway; 6 Norwegian Air Ambulance Foundation, Storgata 33A, Oslo, 0184 Norway; 7 Institute of Clinical Medicine, University of Oslo, Postboks 1072 Blindern, Oslo, 0316 Norway; 8 Mobile Emergency Care Unit, Department of Anesthesiology and Intensive Care, Odense University Hospital, J B Winsløws Vej 4, Odense C, 5000 Denmark; 9 Department of Anesthesiology, Rigshospitalet, Blegdamsvej 9, København Ø, 2100 Denmark; 10 Mobile Emergency Care Unit, Department of Anesthesiology and Intensive Care, Sygehus Lillebaelt Kolding, Sygehusvej 24, Kolding, 6000 Denmark

**Keywords:** Emergency medical services, Major incident, Hazardous substances, Inter-authority cooperation, Preparedness, Triage systems

## Abstract

**Background:**

Major incidents evolving from occupational accidents are very infrequent in Scandinavia and therefore, case reports are called for. On 26 November, 2024, a fatal occupational accident took place during the construction of a concrete silo in the small rural town of Flemløse (population 574), Denmark. Three people died and six were injured as the result of a collapsing concrete roof during construction. We aim to describe the incident response by the emergency medical services (EMS), to identify areas of improvement, and to evaluate the adherence to current national major incident guidelines and communication grids.

**Case presentation:**

The initial call to the emergency medical dispatch center described an accident comprising fifteen injured persons, all of whom were migrant workers. Seventeen EMS units including two helicopter EMS units were dispatched to scene. Three critically injured patients were admitted to a nearby trauma center, whereas three lightly injured were taken to a regional trauma hospital. The initial reports overestimated the number of possible casualties and therefore, the available resources were ample. The very construction of the silo resulted in challenging conditions for evacuation of the injured patients. Chemical, biological, radiological, nuclear, and explosive (CBRNE) aspects of the incident added to the inherent complexity in major incident management. Although potentially detrimental to the patients, the prolonged extrication of the patients enabled the prehospital services to procure a timely organization of the incident site according to guidelines and an organized transport prioritization of the victims. The communication within EMS and between authorities was generally as per national guideline.

**Conclusions:**

The EMS response to this major incident generally adhered to the national guidelines and, furthermore, the communication within and between authorities was established according to guidelines. Important findings included the use of local resources by the incident command and improvised means for the evacuation of victims from a highly hostile environment. The triage of patients adhered to local and national major incident guidelines. Migrant workers have increased risk for occupational accidents.

**Supplementary Information:**

The online version contains supplementary material available at 10.1186/s13049-025-01376-5.

## Background

Major incidents (MI) are defined [1] by the need for more resources than readily available. MI happen infrequently in Scandinavia [2], and even more infrequent are fatal occupational accidents that develop into a MI. However, on a global level, 330,000 people die each year in occupational accidents [3], and every day, approximately 100,000 workers are injured [4]. The scientific literature is scarce and predominantly consists of case reports and non-indexed literature [5]. However, the Danish Trauma Registry [6] provides information on occupational accidents.

In Denmark, there were 29 fatal occupational accidents in 2024 [7]. Except for the presented case, none of them involved more than one fatality. A state-driven authority, the Danish Working Environment Authority [7] is responsible for the area. As the result of the free movement of labor in the European Union [8], a substantive number of migrant workers are stationed in Denmark and other countries around the World. Worldwide, an estimated 170 million people (2021) are working outside their native country [9]. Workers posted to Denmark by a foreign company are covered by a number of rights and obligations, regulated by The Danish Act on the Posting of Workers [10]. Despite these initiatives regulated by law, migrant workers are more prone to be injured or die in occupational accidents for a variety of reasons, including factors such as socioeconomic status, access to training, communication barriers and less rigorous law enforcement due to complex contractual conditions in lack of transparence [11].

We aim to describe the incident response by the emergency medical services (EMS) to a complex MI with difficult patient evacuation and challenging chemical, biological, radiological, nuclear, and explosive (CBRNE) aspects. The objective is to identify areas of improvement, and to evaluate the adherence to current MI guidelines and communication grids.

## Case presentation

### Danish emergency medical services

The entry point for citizens in need of assistance from police, fire brigade, or EMS is the national distress number 1-1-2. Operated by Danish police and Copenhagen Fire Brigade, the operator relays the call to one of five regional emergency medical communication centers (EMCC). The call taker is usually a nurse or a paramedic that utilizes the Danish Index [12], a symptom-based decision-making tool for criteria-based dispatch. The EMS is a three-tiered system comprising approximately 300 ambulances and rapid response units staffed by paramedics and emergency medical technicians. Anesthesiologist-staffed units comprise 24 ground-based mobile emergency care units and four helicopter EMS (HEMS) units. The Danish EMS has been described in detail previously [13, 14].

### Danish trauma system

The Danish trauma system comprises 21 regional trauma units at level 2 and below and four level 1 trauma referral centers located in Copenhagen, Aalborg, Aarhus and Odense. As catchment areas differ between the level 1 trauma centers, the hospitals provide definitive care for 500,000 to 2,800,000 people.

### Major incident preparedness

The guidelines for joint incident command [15] constitute the framework for MI management. The document describes the roles of the authorities involved in a MI, and the cornerstones are sector responsibility, inter-professional cooperation, and the division into the strategic, operational, and tactical levels (Additional file 1). Incident commanders within each sector share the same concepts. They are trained during a mutual three-week course comprising comprehensive education in MI management, extensive radio training and several tabletop and full-scale exercises. Further, authorities are obliged to conduct full-scale exercises at fixed intervals to train and maintain overall MI management skills.

### Major incident communication

The Danish authorities dealing with emergencies, i.e., police, fire & rescue, EMS, some military units and the Danish Emergency Management Agency, use the Terrestrial Trunked Radio (TETRA) standard based system [16]. In the event of a MI, the police issues temporary inter-disciplinary communication channels for mutual use by all three sectors, police, fire & rescue, and the EMS. Further, within the EMS, TETRA channels for direct communication with the EMCC and the hospitals involved are in use during the MI.

### Hospitals in the region

There are five emergency hospitals in the Region of Southern Denmark, of which Odense University Hospital is a Level 1 trauma hospital with a catchment population of the 1,224,000 people.

### Chemical, biological, radiological, nuclear, and explosive – CBRNE - aspects

The MI was the result of an occupational accident that occurred during the casting of a concrete roof of a biogas reactor. Concrete is a composite material, composed of a matrix of *Portland* cement binder and filler aggregate composing of rocky material, loose stones, and sand. The cement binder glues the filler together to form a strong conglomerate. When applied to a cast base, the concrete solidifies and hardens through hydration as water reacts with the cement, which bonds the other components together to form a stone-like material. The chemical reaction for curing concrete is:

$$\begin{aligned}&2\text{Ca}_3\text{SiO}_5+7\text{H}_2\text{O}\cr&\quad\to\:\:3\text{CaO}\cdot2\text{SiO}_2\cdot4\text{H}_2\text{O}+3\text{Ca}\left(\text{OH}\right)_2\end{aligned}$$

Concrete is an alkaline compound that is corrosive to human tissue. Exposure to wet concrete may result in skin irritation, chemical burns and eye irritation. Personal protective equipment includes gloves, boots and eye protection.

### Study design

This case report of the incident is reported as per the CONFIDE (CONsensus guidelines on Reports of Field Observations in Disasters and Emergencies) concept [17] (Additional file 2).

### Data collection

The data sources for the case report comprised the following:

- The electronic Prehospital patient medical record system (Dedalus Healthcare Denmark, Aarhus Denmark): Patient identification, vital parameters, treatment characteristics, time stamps.
- Center of Emergency Communication, Frederiksberg, Copenhagen, Denmark: TETRA communication details, channel selection and affiliation times, push-to-talk messages.
- SimaOffice^®^ control room system (Locus A/S^®^, Aarhus, Denmark), EMCC Region of Southern Denmark, Odense, Denmark: EMS units dispatch time stamps, EMCC physician’s documentation, patient allocation.
- Public domain: Details on roof construction and collapse, contractor details, background.

### Alarm and dispatch

At 18:04, the Danish national distress number 1-1-2 received the first call from the incident site. The call was relayed to the EMCC in Odense at 18:05, a MI was declared at 18:07. Twelve ambulances, three anesthesiologist-staffed mobile emergency care units (MECU), and two HEMS units were dispatched in sequence to the incident site (Table 1). Further, an on-call prehospital physician was summoned to the EMCC as per guideline. The timestamps of events were collected from EMCC control room system.

**Table 1:** Dispatched EMS units to the incident

#	Unit #	Unit name	Arrival at scene	Departure from scene	Arrival at hospital	Released
1	3584	AMB 3584	18:11	-	-	22:06
2	3405	AMB 3405	18:15	18:39	19:26	20:09
3	3554	MECU Odense	18:22			00:03
4	3349	AMB 3349	18:24	19:45	20:04	21:27
5	3523	AMB 3523	18:24	20:47	21:19	22:03
6	3347	AMB 3347	18:25	18:47	19:36	20:02
7	3354	AMB 3354	18:26	19:15	19:37	20:56
8	3563	AMB 3563	18:30	20:03	20:22	22:15
9	3597	AMB 3597	18:33	20:14	21:12	00:12
10	3352	AMB 3352	18:34	20:20	20:51	21:24
11	3555	MECU Svendborg	18:37	20:04	20:22	20:36
12	3365	MECU Kolding	18:48	-	-	20:30
13	3418	AMB 3418	18:48	-	-	20:47
14	3085	HEMS Ringsted	18:53	19:58	20:07	20:19
15	3082	HEMS Skive	19:00	-	-	19:26
16	3500	AMB 3500	19:21	-	-	20:50
17	3415	AMB 3415	19:21	-	-	20:46

Legend: AMB: ambulance; MECU: mobile emergency care unit; HEMS: helicopter emergency medical service

### Scene description

**Fig. 1:**
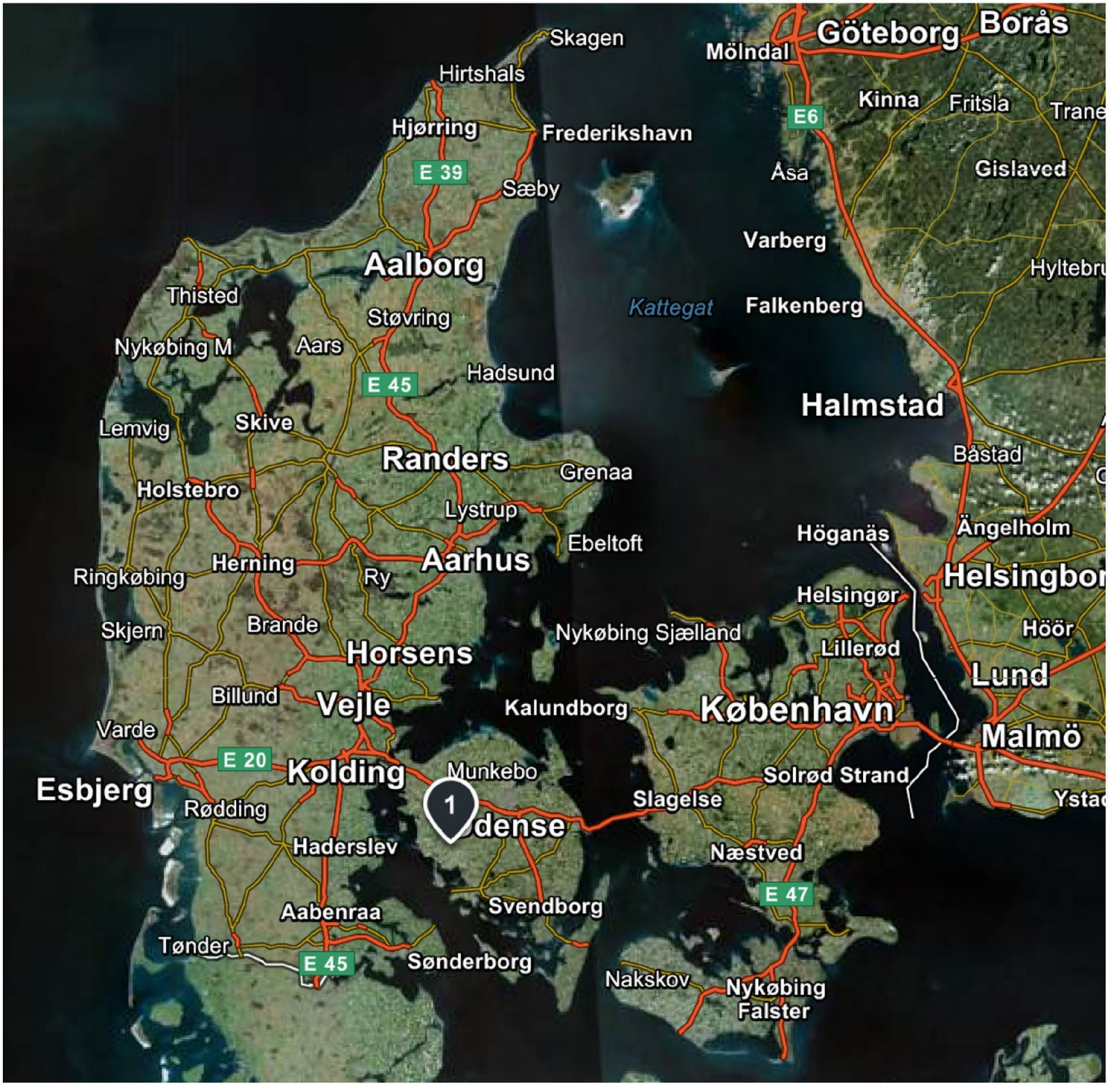
Incident location – map of Denmark

**Fig. 2:**
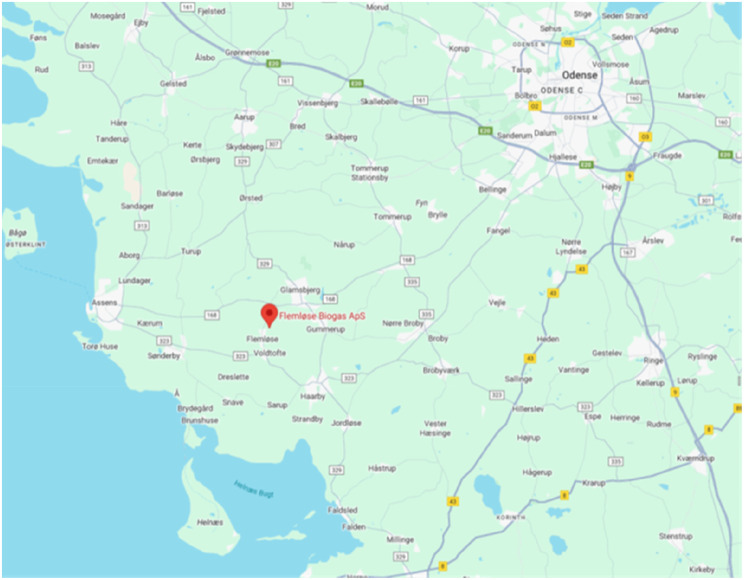
Incident location – local map

**Fig. 3:**
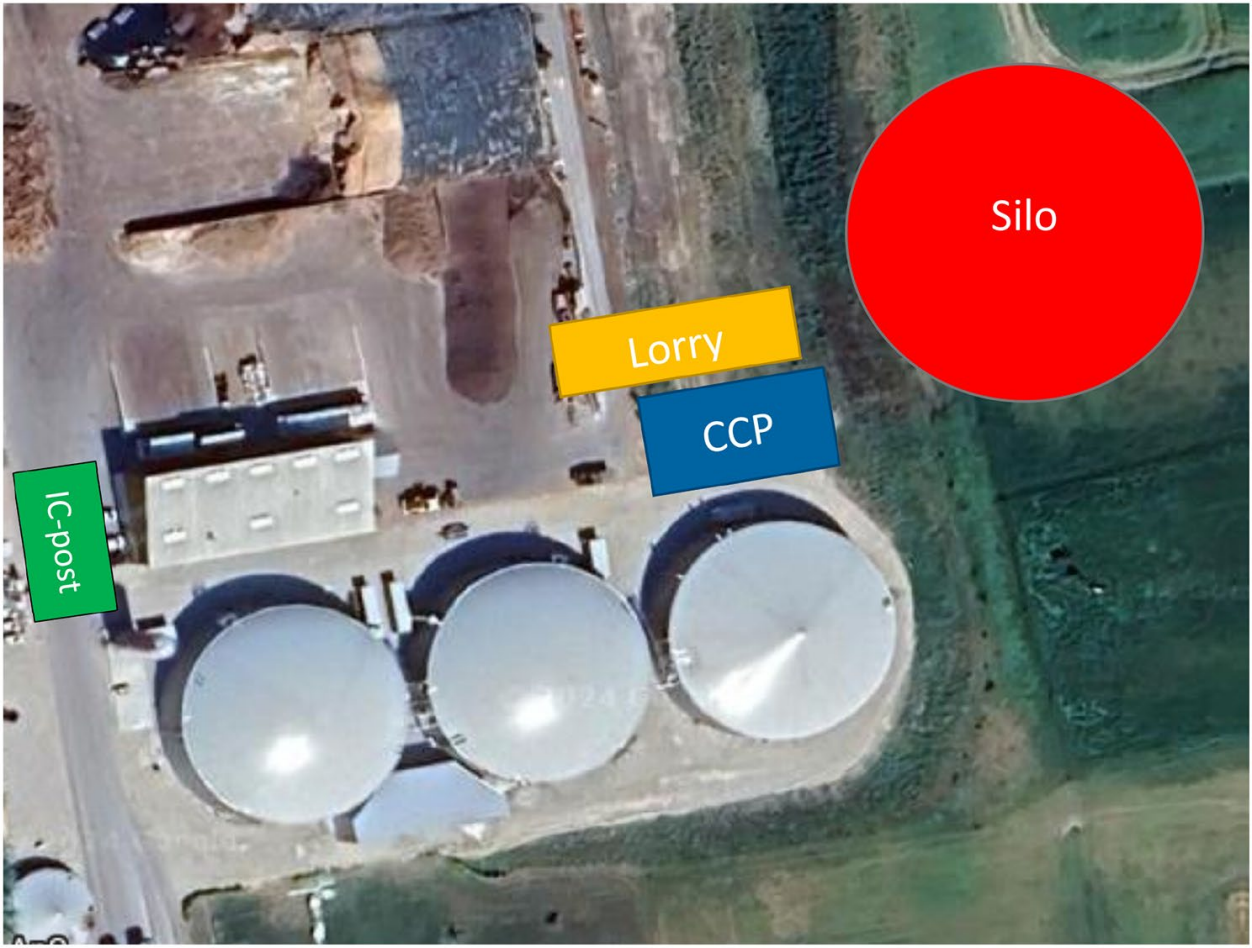
Incident site organization. CCP: Casualty collection point; IC-post: Incident command post

**Fig. 4:**
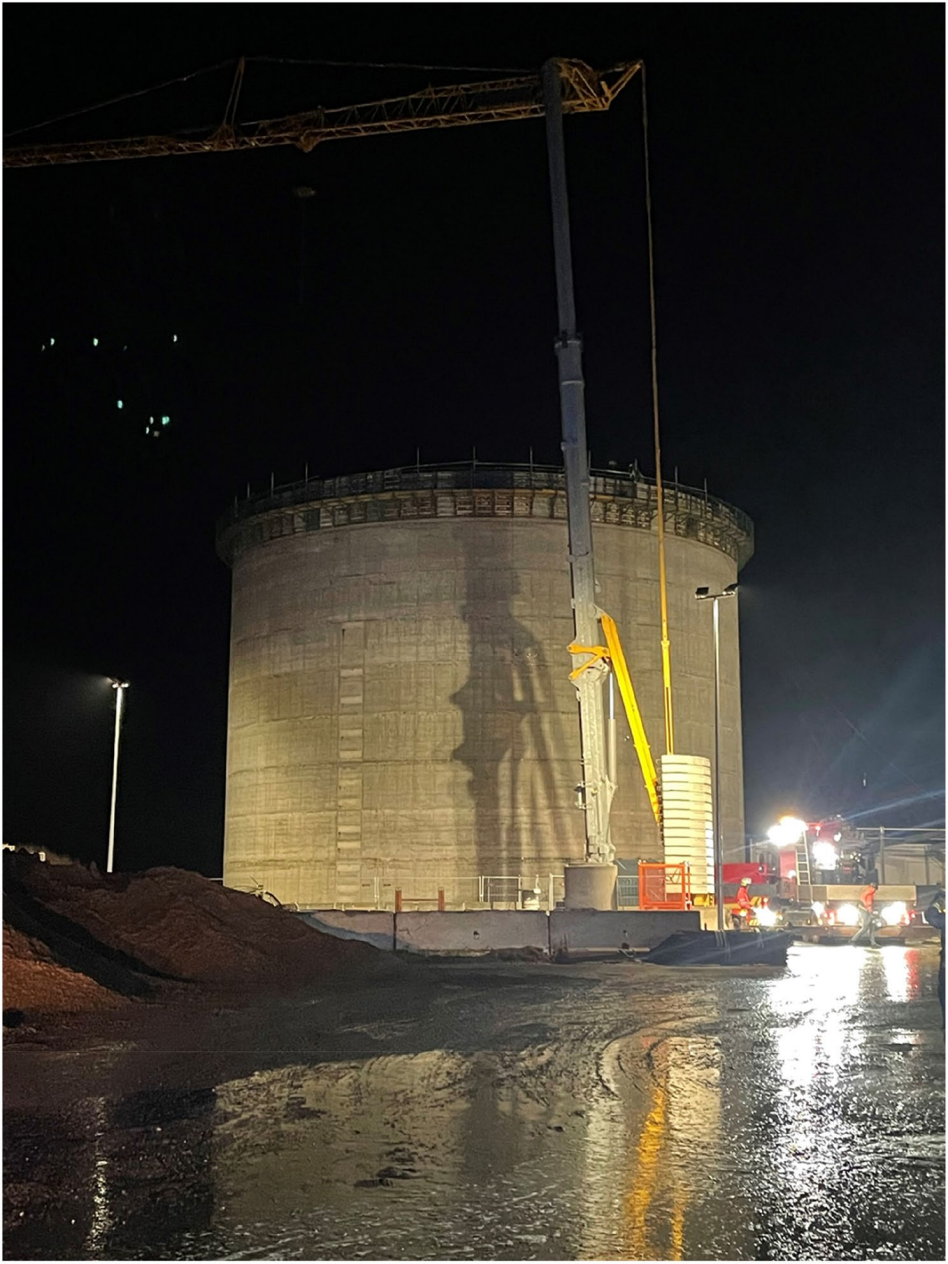
Photo of biogas silo (private photo)

The incident happened in the small rural town of Flemløse (population 574) on the island of Funen (Figs. 1, 2 and 3). A biogas plant was erecting a new silo, a reactor for the oxygen-free production of sustainable energy from agricultural and animal waste. The construction of a circular eighteen-meter-high silo with a diameter of 25 m was in progress, as the concrete roof of the silo was being casted. Preceding the accident, nine construction workers were casting the roof of the silo, using a mushroom-shaped three-legged metal framework (Additional file 3) and a wooden bed for loose concrete propelled from a concrete lorry via a boom to the top of the silo. Inside the silo, there was metal scaffolding at one side of the silo with a staircase to an access hatch at ground level. The silo extended a further ten meters below ground level (Fig. 4).

When approximately 260 of the projected 275 tons of wet concrete had been propelled and distributed to the roof, the metal framework began to give in, descending 3–4 centimeters at one side. The workers immediately halted the work, and made contact to the engineer that had calculated the casting. A few minutes later, the engineer replied, that it was acceptable for the movement and the work continued. Shortly thereafter, the framework collapsed at the westernmost side and the entire structure fell to the ground with the workers trapped in the debris.

On 26 November 2024, at 18:00, the temperature at the incident site was 7.4 degrees centigrade with precipitation at 0.1 mm and the middle wind speed at 6.7 m/sec.

### Site access and security

There was one access route to the site approximately 300 m from the silo. This access route was declared as the ambulance staging area. The fire brigade relayed information to EMS that the danger zone (the interior of the silo) was unsafe and not accessible to EMS as access was restricted to fire personnel using personal protective equipment such as helmets, boots and gloves. When the fire brigade permitted access, the medical MI commander decided to position one of the five prehospital physicians and his paramedic assistant inside the silo to conduct immediate treatment and stabilization of critically injured patients before evacuation from the silo. Prior to entry, two persons had evacuated themselves. One additional person was evacuated via the scaffold and hatch supported by fire personnel.

After removal of the large concrete transport lorry, a mobile crane that happened to be at the site, was commissioned by the authorities to hoist patients who were unable to walk or deceased. They were hoisted from the silo in basket stretchers after initial treatment and stabilization inside the silo. Inside the silo, the environment presented significant challenges. Debris consisting of wood and metal from the collapsed roof piled up nearly ten meters high, with an additional layer of 40–50 cm of floating concrete, obstructing access to the remaining individuals. This made patient access and mobility exceedingly difficult.

### Site organization

According to national MI guidelines, the danger zone was defined to be the silo. Inner and outer cordons designated the incident scene. The police guarded and controlled the access to the incident site (Fig. 3). The police positioned one vehicle as an incident command post for documentation and for meetings for the joint incident command. Police designated two landing sites for HEMS, to the north and south of the incident site.

An interim casualty collection point (CCP) was set up next to the silo access hatch, with easy ambulance access via the staging area. The ambulance personnel placed supplemental oxygen, blankets, and bags with medication at the CCP. Further, the site company supplied a six by six meter tarpaulin as the ground had a muddy yet firm surface. Fire service set up sufficient interim floodlights. Inside the silo, the fire and EMS crews used headlamps.

### Medical incident command

The first MECU physician to arrive was designated Medical Incident Commander (MIC). The MECU paramedic assistant was designated radio operator to free up resources for the MIC´s coordination efforts. Shortly after, the commanders of fire & rescue and police arrived, and the Joint Incident Command (JIC) was formally established. Rescue personnel inside the silo briefed the JIC by TETRA radio, reporting that three individuals had evacuated themselves via the stairs and the silo exit at ground level. Within the silo, three individuals were awake but severely injured, and three appeared lifeless.

The situation was further complicated by the presence of a concrete lorry, which had been pumping concrete onto the silo roof and was still obstructing access for heavy machinery. It was estimated that it would take forty minutes to move the vehicle.

When the second MECU physician arrived on-site, the physician was immediately assigned to casualty clearing officer.

The JIC held its first interdisciplinary meeting approximately twenty minutes after arriving on-site. The police informed the team that nine individuals were believed to have been on the silo roof when it collapsed. The fire services confirmed that the scene was safe at this point when wearing a helmet, but emphasized challenges with evacuating casualties through the scaffolding connected to the ground-level service hatch.

After the site was declared safe, the MIC, in consultation with the casualty-clearing officer, deployed a medical team consisting of one EMS physician and one physician’s assistant into the silo for initial treatment. As soon as the individuals were evacuated outside the silo, they were registered in the Prehospital patient medical record system (Dedalus Healthcare Denmark, Aarhus Denmark) [14] and were reassessed by the casualty clearing officer for triage. The EMCC physician was continuously updated on the progress of the evacuation, each casualty’s condition and suspected injuries.

The EMCC physician was able to determine in advance which hospital in the region each individual casualty should be transported to. The JIC convened approximately every 15–30 min, conducting a total of four meetings during the operation to coordinate updates and ensure efficient collaboration across teams.

### TETRA communication

As per guideline, a default joint incident command channel was relayed, and police issued special designated interdisciplinary channels. Data collected from the logs acquired from Center of Emergency Communication display that there were 35 active radios in use by EMS in the incident and 14/17 (82.4%) of the dispatched EMS units switched to the designated channels (Additional file 4 and 5). There were 466 push-to-talk messages transmitted by EMS during the incident. The non-standard shifts were primarily to the default joint incident command channel by units not authorized to that channel.

### EMS resources

**Fig. 5:**
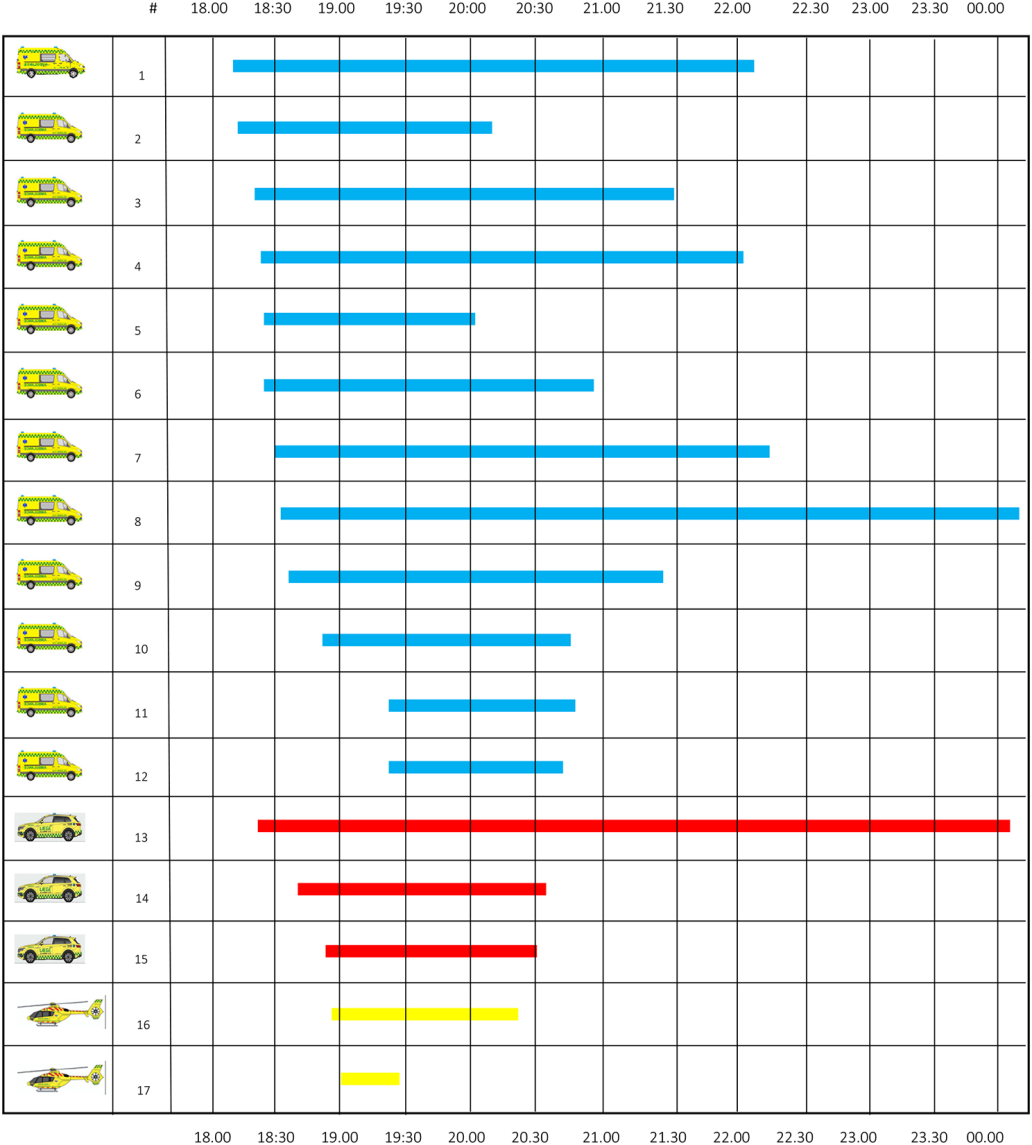
Overview of units and time expenditure

The records of the control room system display that the EMCC promptly dispatched eight ambulances, three on-duty MECUs and two helicopter EMS units. A further four ambulances were dispatched to the incident (Table 1; Fig. 5). The casualty clearing station officer quickly released one helicopter and sequentially, ambulances were released by the incident command, to handle regular ambulance missions in the region. A total of four ambulances and one MECU was commissioned from the neighboring regions for tasks not related to the MI.

### Initial treatment

The medical team conducted initial triage, and emergency treatment was initiated for three patients found alive at the scene. Two additional individuals were discovered partially buried under the concrete, one of whom exhibited extensive physical trauma; both were pronounced life extinct at the scene. One individual remained unaccounted for at the time.

Due to the hazardous conditions and logistical challenges, monitoring equipment was initially not prioritized for treatment inside the silo. Triage and initial assessments were conducted manually despite impaired visibility, using helmet-mounted lamps. The patients, partially covered in concrete, presented themselves with severe injuries. Two individuals were alert, with severe pain in the back, torso, abdomen, and extremities, whereas the third had suspected lower extremity fracture and reported significant pain.

The emergency treatment included the administration of intravenous analgesics such as ketamine and fentanyl, as well as antifibrinolytics and supplemental oxygen.

The narrow scaffolding and small hatch made it impossible to evacuate the patients by conventional means. The first and most critical patient of the three patients unable to walk was evacuated through the access hatch, using ropes and a basket stretcher. The next two patients were hoisted by crane, ascending approximately 30 m (Additional file 6). The evacuation process for all three injured individuals required approximately 45–60 min due to the complex and constrained environment. Later on, the on-scene crane also hoisted the two life extinct individuals.

While waiting for evacuation, the patients were closely monitored and treated by the medical team with support from firefighters. Non-invasive blood pressure and pulse rate were measured using standard monitoring equipment, which was later transported into the silo. However, due to concrete contamination of the skin, oxygen saturation measurements could not be obtained. The preliminary patient registration was initiated using the electronic patient medical record system [14].

The first patient evacuated via ropes was the most critical, exhibiting a progressively deteriorating Glasgow Coma Scale score during the rescue operation.

Working in the floating concrete significantly complicated patient treatment and movement between patients. All surviving patients remained relatively stable, despite delayed evacuation.

### Triage

The six patients admitted to hospitals were triaged by the casualty collection station officer, using Triage Sieve and given a transport priority and destination hospital as per EMCC physician’s discretion. Triage category was documented in the electronic prehospital patient record system.

### Secondary treatment and patient characteristics

A total of nine patients were present as the roof of the silo collapsed and fell approximately 28 m to the ground along with debris such as metal casting, wooden roofing and wet concrete. Three patients died in the incident; three were critically injured from primarily blunt mechanism, suffering limb fractures and internal bleeding, admitted to the nearby trauma center, 28 km away. Three were lightly injured and admitted to a regional trauma unit, located 69 km from the incident site. All six patients received treatment en route to hospital.

The mechanism of injury was blunt force trauma. Treatment consisted of supplemental oxygen, large bore intravenous cannulae, intravenous fluid, and pain medication with opioids as indicated. Prehospital physicians accompanied two of the critical patients. Further, the patients suffered from light chemical burns and corneal abrasions due to the chemical properties of the curing concrete.

### Patient distribution to hospitals

The physician stationed in the EMCC distributed patients to two hospitals, based on the on-site triage and on a short verbal report from the ambulance officer. The digital prehospital patient record system enabled a total overview of the patients, their injuries, the individual ambulance and the destination hospital. For identification and documentation of the victims of the MI, police stationed officers at the receiving hospitals upon arrival of the patients.

### Hospital preparedness

Based on the initial reports of the incident, the EMCC physician informed two hospitals in the region to prepare for mass casualty protocol activation as per preparedness plans. The trauma center at Odense University Hospital activated a mass casualty protocol, as six to ten patients were expected (Additional file 6). Kolding Hospital trauma unit also activated their mass casualty protocol.

The EMCC physician communicated with the receiving hospitals through a digital tool that provides information on the sex, age, and suspected injuries of the incoming patients. Further, a wristband applied prehospitally acted as a unique patient identifier. The EMS relays this unique patient identifier to the hospitals. Formal identification takes place in the hospital, if possible. In this incident, police was present in the hospital, as the patients were all foreigners from different European Union countries.

### Local operational staff

Local operational staff (Additional file 1) was activated at 20.00, and remained in session for the duration of the search for the missing person, i.e., four days. Representatives from all involved authorities and the local municipality assembled at the police district headquarters in Odense. The authorities coordinated the continued search and rescue effort, published press releases and conducted doorstep interviews regarding the accident.

### Continued assessment

As the living patients were transported to hospitals for treatment, a further two patients were pronounced life-extinct at the scene by a prehospital physician. Further, in the following days, the search for a missing person continued using the Urban Search and Rescue – USAR - capabilities of Danish Emergency Management Agency. The deceased individual was located after three days (Additional material 7).

### Defusing and debriefing

At the incident site, the driver of the concrete lorry was in emotional distress due to the event, and a psychological debriefing team was commissioned by the police as per guidelines. No further patients or bystanders were considered in need of defusing.

Immediately after the incident, a defusing took place at the ambulance base in Odense, conducted by the MIC and the ambulance officer. Two MECUs and five ambulance crews were able to participate in the meeting. There was a detailed run-through of the events and the EMS performance by the medical directorate of the Region of Southern Denmark, based on the written feedback from all personnel involved in the MI.

## Discussion

### Challenges encountered by EMS in the concrete silo accident

#### Site access and security

The access road to the incident site was a small rural country road. However, it still left space to function as the ambulance staging area, allowing sufficient access for additional emergency units to pass. The CCP was positioned outside the access opening into the silo. As and when patients were evacuated from the silo, ambulance crews and the casualty clearing officer received the patients, performed triage and made transport prioritization upon departure of the ambulance to the designated receiving hospital. Patient identification numbers and vital signs were reported back to the EMCC physician who relayed the information to the receiving hospitals for shared situation awareness.

#### TETRA communication

The communication within and between the emergency authorities was successful at 82.4% in this particular incident, contrasting previous MI in Denmark [18, 19]. Communication difficulties are predominant in sudden-onset MI for a multitude of reasons [20], including factors such as interface design, the startle effect, and lack of initial training [21]. The successful communication in this MI is the result of an effort within EMS to improve TETRA communication in general and MI specifically. Improved and focused initial training and continued frequent micro training in the TETRA radio have raised awareness and contributed to a documented substantively improved performance.

#### EMS resources

Because of the declaration of an MI within the EMCC, based on the initial reports from the incident site, the EMS resources were sufficient. As per the action principle in Danish crisis management [22], the immediate response to the MI was prompt and over triaged, as it turned out that the number of victims from the incident was lower than first expected. However, ambiguous reports on the precise number forced the EMCC dispatch to allocate sufficient EMS units to accommodate the suspected number of victims.

#### Emergency communication center perspective

In the EMCC, an on-call prehospital physician was present as per MI guidelines with the task of allocating patients to hospitals from the incident site. The allocation of patients was based on prehospital triage by the casualty clearing station officer and from detailed knowledge of suspected injuries and hospital capacities.

Furthermore, the EMCC physician and the dispatch personnel decided to request assistance from two neighboring regions for additional ambulances to maintain preparedness to everyday regular patients. In similar MI [18, 19, 23–24], the presence of a EMCC physician adds to the overall EMS situation awareness that is paramount in MI management, to maintain resilience for routine medical tasks.

#### Ambiguous initial reports

From the initial report, an estimated ten to fifteen persons were involved in the silo accident. The report was based on the information received by the 1-1-2 central from a caller at the incident site. During the course of the incident management, there were discrepancies regarding the precise number of patients involved and considered injured in the accident. The numbers provided by the owner of the plant were higher than those from the representative of the company employing the workers at the site. This led to confusion in the incident command and the treating personnel as to the exact number of casualties from the incident site. This confusion hampered and prolonged the search for persons trapped under the debris at the bottom of the silo.

#### Patient treatment and characteristics

The patients were all male migrant workers. The patients suffered injuries and blunt trauma from the fall and chemical burn from exposure to concrete.

The prehospital treatment consisted of intravenous access, analgesics such as Ketamine and Fentanyl, Tranexamic acid, and the splinting of suspected pelvic fracture. Further, the contamination with concrete left some of the patients with injuries to skin and eyes. In these cases, the prioritization to load-and-go prevented further decontamination. This was decided by the treating physicians in this MI because of resource shortage, as fire personnel, who usually perform decontamination, was occupied by evacuation from the silo. However, the receiving hospitals have the capacities for patient decontamination as per mass casualty protocols.

#### Chemical, biological, nuclear, radiation, explosives aspects

The complexity of the MI was substantively enhanced by the CBRNE aspect of the alkaline chemical properties of the concrete. The safety of the patients and EMS personnel was to some extent compromised, as life-saving evacuation and urgent transfer to final treatment was the priority of the treating physicians and the incident command. The patients suffered from varying degrees of injuries because of exposure to curing concrete. CBRNE represents competence gaps in MI management [25] and optimizing training methods for different CBRNE situations are called for. MIs involving CBRNE are rare [26–27] and challenges EMS even further [28], as both patient needs of advanced treatment and the protection of hospital personnel put a strain on resources and require resilience.

#### Hospital preparedness

Based on the expected number of potential patients to be admitted to the trauma center at Odense University Hospital, it was decided by the hospital preparedness management to activate the second tier of the mass casualty protocol. When the exact number of casualties scheduled for the trauma center became known, it was decided to de-escalate to a lower level. Similarly, the regional trauma unit activated their trauma mass casualty protocol.

Hospital preparedness is of major importance to mitigate the effects of MI [24, 29, 30] on hospital surge capacity [31], and the ability to triage and treat critically ill patients along with the regular intake. The task forces hospital administrators to produce updated preparedness plans, and Odense University Hospital published a new plan [32] prior to the incident on 26th of November 2024 (Additional file 7).

#### EMS major incident medical preparedness

As MI preparedness is pivotal [25, 33–34], the EMS in the Region of Southern Denmark specifically prepares and trains the response to MI. Preparations include strategic preparedness planning, crisis management, and cooperation and coordination with other emergency authorities. The command structure analogous to Gold—Silver—Bronze or Strategic – Operational – Tactical levels [15] within the EMS organization has been effective for a more than a decade (Additional file 1).

Post-hoc evaluations of EMS performance in MI [35] may give rise to new management guidelines, and the training of MI in full-scale exercises are re-introduced post-COVID-19. The challenge with exercises has been described elsewhere [36]. As gaps between exercise scenarios and real-life MI do exist, the study suggests that the training and personnel preparations should reflect the complexity of real-life incidents for the incident command to be successful.

#### Migrant worker safety

The safety of migrant workers is a worldwide problem [6, 9, 37–38], echoed in this case report with nine migrant workers killed or severely injured in an occupational accident that developed into a MI. The benefits of free labor movement in the European Union [8] are challenged by the fact that while migrant workers comprise 12–13% of the labor force in Denmark, they accounted for 37% of fatal occupational accidents [39] between 2016 and 22. The report emphasizes lack of basic human and occupational rights. Further, the insufficient safety measures in employment and work execution contribute to an unjustified risk of injury and death for migrant workers. The authors of that particular report suggest that national authorities enhance legislation and control on *chain liability* in business to mitigate the effects of lacking transparency in responsibility. The problem is mirrored in this case report, as the exact number of workers on the site was unreliable.

In a systematic review and meta-analysis [11] comprising 44,338 migrant workers, the authors concluded that migrant workers have a higher risk of workplace fatal injury. Contrasted by being generally healthier than local workers, the risk of fatalities may be explained by structural factors such as inadequate safety protection and precarious employment. In another study [40, 41] of 12,168 migrant workers, the authors find that migrant workers are at substantive risk of work-related injuries and ill health, as the health needs of migrant workers are substantively overlooked in research and national policies. The authors suggest that authorities and businesses should enforce and improve occupational health and safety measures. Finally, in a review of migrant workers’ use of health services [41], the authors found that migrant workers may be less likely than non-migrant workers to use health services and more likely to have occupational injuries due to factors such as illegal migration, different migration stages, migration irregularities, and the informal economy [42, 43], that is inherent in some aspects of migrant work.

#### Lessons learned

The MI that evolved from an occupational accident involving ten migrant workers was generally managed according to the national guidelines. Lessons learned from this case report for future improvement include:

- The need for exact information about the number of patients involved in an accident should be precise and updated. The use of local resource persons is paramount to obtaining reliable information.
- The possible CBRNE aspects in MI should be addressed in MI incident command and efforts to mitigate subsequent injury from exposure should be discussed and prioritized against life-saving procedures.
- The use of ad hoc capabilities such as a crane that happened to be at the site should be in the MI guidelines and taught at courses and exercises.
- The sustainment and relief of personnel is important and should be in the contingency plans by the incident command.
- Updated hospital mass casualty protocols are pivotal to ensure surge capacity in MI.
- An experienced prehospital physician in EMCC is important for continued assessment, allocation of patients and the return to a normal state if EMS.

#### Strengths and limitations of the study

The strengths of this case report include data consistency and access to all relevant data from the EMS. Most of the authors had predominant roles in the MI and therefore, the case reports constitutes an almost complete picture of the events in the MI described. There are several limitations as well. These include recall and selection bias inherent in observational studies, since there is no causality between the EMS response and patient outcome. However, the findings of the case report are possibly generalizable and transferable to prehospital organizations in similar geopolitical arenas.

## Conclusion

The silo accident on 26th November 2024 in Denmark was a complex and fatal occupational accident that evolved into a MI. Seventeen EMS units were dispatched to the construction site which was extremely difficult to access for rescue services. Furthermore, the CBRNE aspects - the exposure to curing concrete - challenged the medical incident command. The performance of the EMS generally adhered to national guidelines and benefitted from substantive resource allocation, resilience and training. Important findings included difficult access to the site of the accident requiring improvised actions to evacuate the patients trapped within the silo. Other important findings include successful TETRA communication and our finding that all the injured patients were migrant workers, underscoring their increased risk of injury in occupational accidents.

## Electronic supplementary material

Below is the link to the electronic supplementary material.

Supplementary Material 1

## Data Availability

No datasets were generated or analysed during the current study.

## Abbreviations

CBRNE: Chemical, biological, radiation, nuclear, explosives
CCP: Casualty collecting point
EMCC: Emergency medical communication center
EMS: Emergency medical services
HEMS: Helicopter emergency medical service
MECU: Mobile emergency care unit
MI: Major incident
MIC: Medical incident commander
TETRA: Terrestrial trunked radio
