# Supplementary material for: Concrete silo collapse: emergency medical services response to a mass casualty incident
Source: Scand J Trauma Resusc Emerg Med. 2025 Apr 7;33:58. doi: 10.1186/s13049-025-01376-5 (PMC11977906; doi:10.1186/s13049-025-01376-5)

**Additional files**

Additional file 1. Danish National Crisis and Major Incident Management System (from Hansen et al.:

The Great Belt train accident: the emergency medical services response).

Additional file 2. CONFIDE checklist. Quality assessment framework of non-traditional study type.

Additional file 3. Drawing of the roof construction.

Additional file 4. List of TETRA radio shifts by ground-based units

Additional file 5. TETRA talk group assignments.

Additional file 6. Odense University Hospital mass casualty plan.

Additional file 7. Pictures from the inside of the silo and incident site.

**Additional files**

Additional file 1.Danish National Crisis and Major Incident Management System (from Hansen et al.: The Great Belt train accident: the emergency medical services response).

Government Security Committee

**STRATEGIC LEVEL - GOLD**

Senior Officials Security Committee

International Operational Staff

National Operational Staff

**OPERATIONAL LEVEL - SILVER**

Local Operational Staff

**
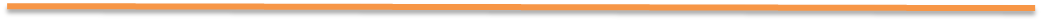
**

**TACTICAL LEVEL - BRONZE**

Local Incident Command

Police

Fire & Rescue

EMS

Ambulance Incident Commander

Casualty Clearing Station Officer

Emergency Medical Commuication Center

Ambulance

Ambulance

HEMS

SAR/JRCC

Legend: EMS: Emergency medical services; HEMS: Helicopter emergency medical services; SAR: Search & Rescue; JRCC: Joint rescue coordination center

Additional file 2. CONFIDE checklist

Quality assessment framework of non-traditional study type.

**Robustness: study information and context**

**Score Time period of study/data collection Definition**

A Clearly defined: Exact time frame of data collection given

B Some definition: Approximate time frame given

C No definition: No time frame given

**Score Sample population Definition**

A Clearly defined: 3 out of 4 parameters from age, gender,

number and study population taken from

B Some definition: 1 or 2 parameters defined from above list

C No definition: No parameters defined

N N/A: Not applicable - none population study

**Score Disease description/patient condition Definition**

A Clearly defined

B Some definition

C No definition

N N/A

**Score Author bias Definition**

A Clearly defined Clear who the author is writing on behalf of

with some reflection of potential bias

B Some definition Clear who the author is writing on behalf of

with minimal reflection of potential bias

C None Either not clear who the author is writing behalf of or

clear but with no acknowledgement of potential bias

**Score Type of language Definition**

A Minimal Factual; less than 10% emotive language

B Low bias Less than 30% of language includes below

C Moderate bias 30 - 50% of language includes below

D High bias Greater than 50% emotive language used: use of

"I"; personal beliefs, reflections or experiences;

inner experiences

**Generalizability**

**Score Author perspective Definition**

A The study was written and reported in country by a

native of the country.

B The study was written and reported in country by an

expatriate working as part of the response.

C The study was written and reported externally (to

the country) by an expatriate working as part of or

observing the response.

**Score Applicability Definition**

A Very applicable Results/findings are applicable, relevant and likely

to be similar in other settings

B Moderately applicable Results/findings are applicable, relevant and likely

to be similar in other settings but some aspects are

specific to the study setting only

C Low applicability Results/findings are only applicable and relevant to

the study setting and not likely to be similar in other

settings.

N N/A Opinion piece

**Added value**

**Score Lessons learned Definition**

A High System/organisational level lessons learned,

described and discussed

B Moderate Individual lessons learned

C Low None or limited lessons learned

**Score Triangulation to the literature Definition**

A High Findings linked to previous studies, guidance or

literature as part of discussion

B Moderate Some linkage to previous studies, guidance or

literature

C Low No linkage to previous studies, guidance or

Literature

**Score Implications Definition**

A Good Contributes something new and/or different in

terms of understanding/insight or perspective;

suggests ideas for further research; suggests

implications for policy and/or practice

B Fair 1 or 2 of the above

C None None of the above

**Ethics**

**Score Ethics Definition**

A Good Where relevant ethical consideration has been

discussed and approval gained (e.g. in use of photos, patient identifiable data, informed consent)

B Fair Some mention of ethical considerations but no clear

ethical approval sought or gained

C None No mention of ethical consideration; unclear if

permission sought or gained

N N/A Not relevant/none population study

Additional file 3. Drawing of the concrete roof construction (From Arbejdstilsynet).


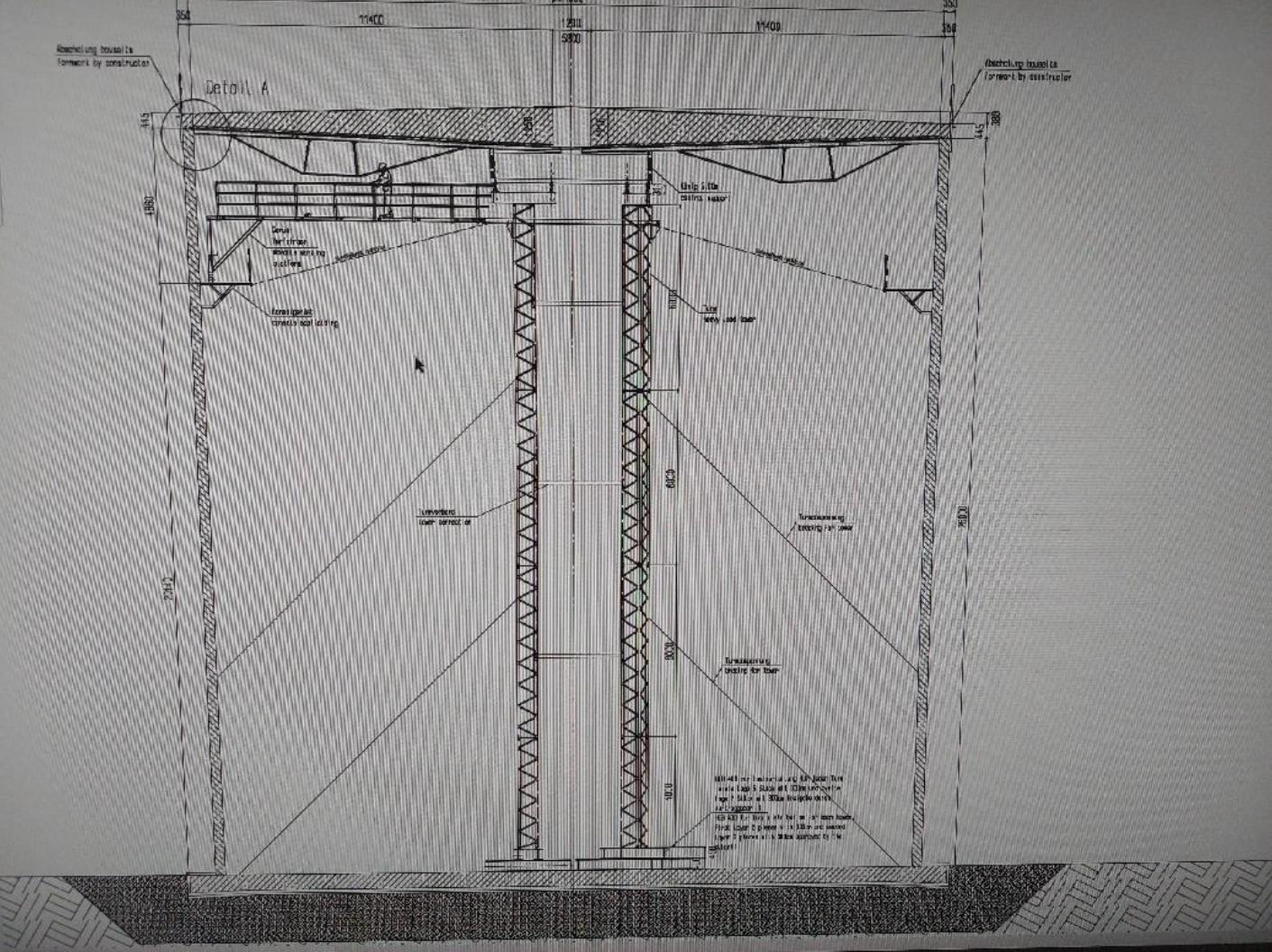


Additional file 4. List of TETRA radio shifts by ground-based units

Additional file 5. TETRA talk group assignments.

| **Unit** | **Talk groups as per guideline** | **Remarks** |
| --- | --- | --- |
| AMB 1 | IC60, HEALTH 61 | Correct |
| AMB 2 | HEALTH 61 | Correct |
| AMB 3 | HEALTH 61 | Incorrect, also in IC60 on arrival |
| AMB 4 | HEALTH 61 | Mainly correct, shortly in IC60 before arrival |
| AMB 5 | HEALTH 61 | Mainly correct, shortly in IC60 before arrival |
| AMB 6 | HEALTH 61 | Correct |
| AMB 7 | HEALTH 61 | Incorrect, also in IC60 on arrival |
| AMB 8 | HEALTH 61 | Incorrect, also in IC60 on arrival |
| AMB 9 | HEALTH 61 | Mainly correct, shortly in IC60 before arrival |
| AMB 10 | HEALTH 61 | Correct |
| AMB 11 | HEALTH 61 | Correct |
| AMB 12 | HEALTH 61 | Correct |
| MECU 1 | IC60 -> IC61, HEALTH 61 | Correct |
| MECU 2 | IC60, HEALTH 61 | Correct |
| MECU 3 | IC60, HEALTH 61 | Correct |
| HEMS 1 | IC60, HEALTH 61 | Correct |
| HEMS 2 | IC60, HEALTH 61 | Correct |

Legend: AMB: Ambulance; MECU: Mobile emergency care unit; HEMS: Helicopter EMS; IC: Incident command

Additional file 6. Regional major trauma center mass casualty plan (surgical).

| Level 1 | 3-5 casualties w/ major injuries |
| --- | --- |
| Level 2 | 6-10 casualties w/ major injuries |
| Level 3 | More than 10 casualties w/ major injuries and/or more than 50 casualties w/ moderate injuries |

Additional material 7. Pictures from the inside of the silo (private photos).


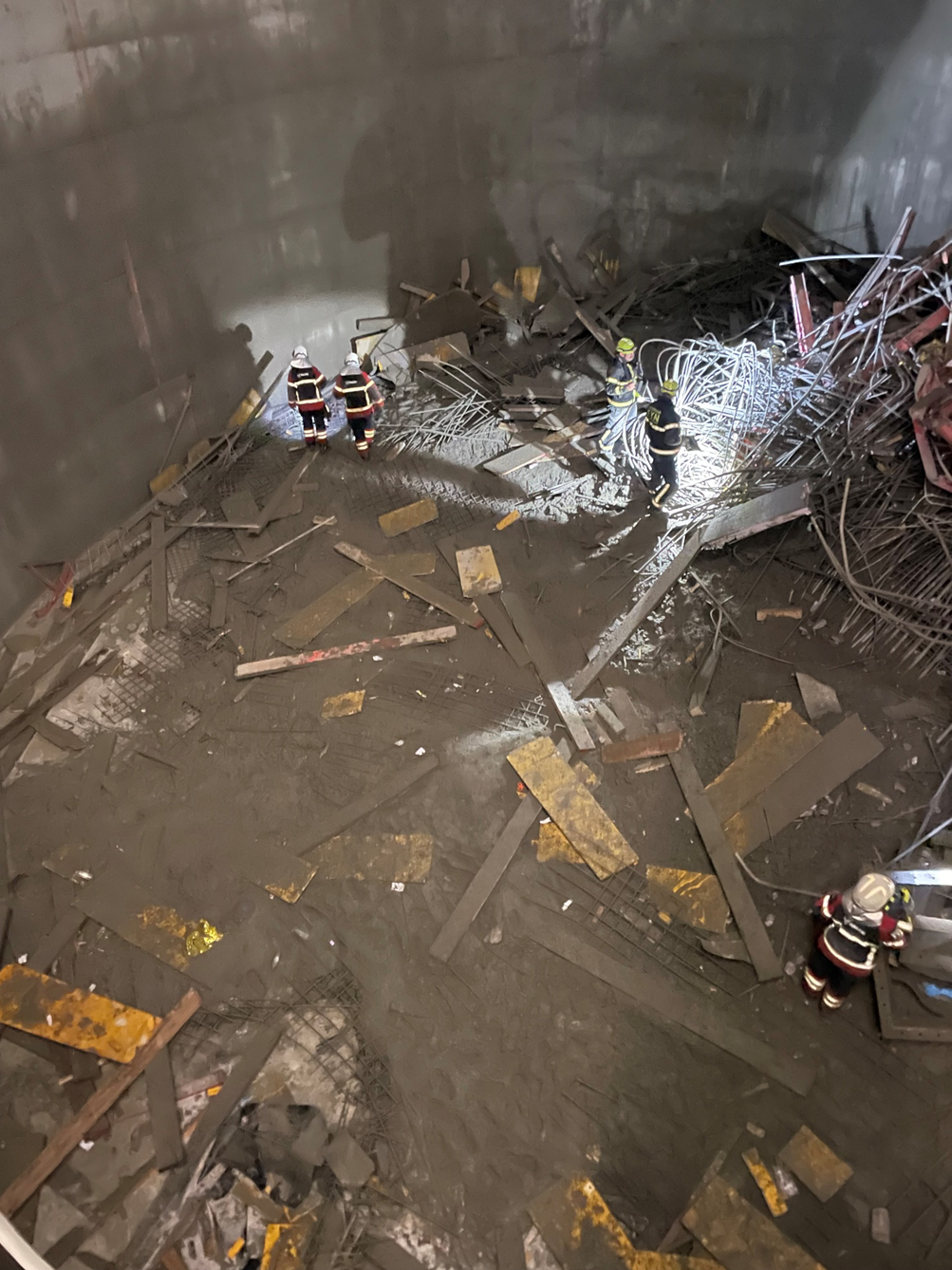


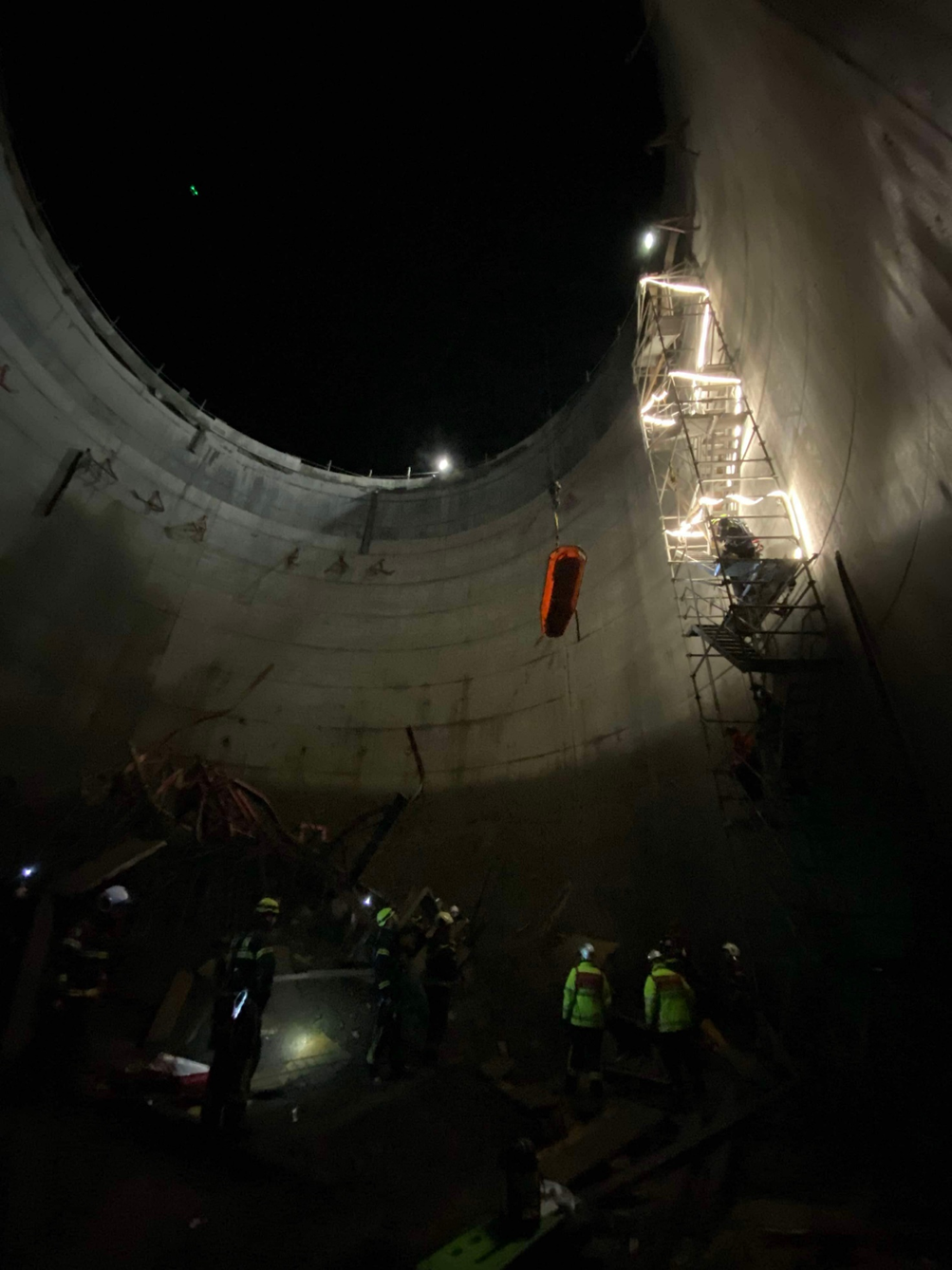

Supplement: Supplementary file 1 — Supplementary Material 1 [file 13049_2025_1376_MOESM1_ESM.docx]
